# Hypothermia after decompressive hemicraniectomy in treatment of malignant middle cerebral artery stroke: comment on the randomized clinical trial

**DOI:** 10.1186/s13054-019-2452-3

**Published:** 2019-05-09

**Authors:** N. Engrand, M. Mazighi, V. Dinkelacker

**Affiliations:** 1 Neuro-Intensive Care Unit, Fondation Ophtalmologique Rothschild, 29 rue Manin, 75019 Paris, France; 2 Interventional Neuroradiology Department, Fondation Ophtalmologique Rothschild, 29 rue Manin, 75019 Paris, France; 3 Neurological Department, Fondation Ophtalmologique Rothschild, 29 rue Manin, 75019 Paris, France

Therapeutic hypothermia (TH) is currently evaluated in acute brain injury of diverse etiologies. In the clinical trial DEPTH-SOS, Neugebauer et al. [1] reported negative results for TH (33 ± 1 °C) in malignant acute ischemic stroke (AIS) in adjunction to decompressive hemicraniectomy. TH improved neither survival at day 14 or at 12 months, nor long-term functional outcome. Instead, severe adverse events (SAE) in the TH-group entailed premature trial discontinuation. We revisit the study design with regard to its clinical objectives.

TH may have two objectives in acute brain injury: intracranial pressure (ICP) control or neuroprotection. Regarding ICP control, it is the relative hypocapnia due to hypothermia which allows an ICP decrease, and hypocapnia will therefore be tolerated (alpha-stat principle) [2]. For the aim of neuroprotection, hypothermia-induced hypocapnia is counterbalanced with respirator settings modification (minute volume decrease) in order to restore normocapnia (pH-stat principle) [3]. Accordingly, studies assessing neuroprotection while capnia management was based on the alpha-stat principle have shown no positive effects [4].

In the case of AIS, the choice of either of these strategies depends on the clinician’s objective.

Indeed, the alpha-stat management allows ICP control and thus prevents reperfusion hyperemia as a source of hemorrhagic transformation, while the pH-stat management ameliorates neuroprotection in the penumbra area.

In the DEPTH-SOS trial, TH was performed to obtain ICP control as adjunctive therapy to decompressive hemicraniectomy, but failed to prevent early mortality from malignant AIS.

In our opinion, a major reason for this failure is that PaCO2 was obviously managed according to the pH-stat principle (although it is not specified), since ICP was superior in the TH-group compared to the control group during the TH period. The pH-stat management may have also contributed to excess SAE in the TH group, since it induces relative hypercapnia (given the hypothermia) and therefore increases reperfusion hyperemia with its inherent deleterious effects.

Finally, TH was effective rather late, over 48 h after AIS and most often after vascular recanalization, i.e., with a reperfused penumbra area. In this scenario, the pH-stat management has only limited impact on neuroprotection.

After an AIS reperfusion, it is crucial to control PaCO2 and arterial blood pressure to closely match cerebral blood flow (CBF) to oxygen requirements. The goal should be to provide the minimum CBF needed in healthy territories, in order to limit hyperemia in infarcted territories, through suitable monitoring (transcranial Doppler, or brain tissue oxygen partial pressure measurement).

In conclusion, this study pools patients with different characteristics of hemodynamics and brain injury, which limits the interpretation of the findings. An individually targeted TH approach, based on carefully monitored cerebral hemodynamics, may be more appropriate for AIS patients. Hence, TH should not be systematically discarded in AIS, but its management should be adapted to the individual patient’s profile. This study illustrates once more how neuroprotective strategies fail due to inappropriate timing and treatment objectives.
